# A Novel Mobile Health App to Educate and Empower Young People With Type 1 Diabetes to Exercise Safely: Prospective Single-Arm Mixed Methods Pilot Study

**DOI:** 10.2196/29739

**Published:** 2021-10-14

**Authors:** Vinutha B Shetty, Wayne H K Soon, Alison G Roberts, Leanne Fried, Heather C Roby, Grant J Smith, Paul A Fournier, Timothy W Jones, Elizabeth A Davis

**Affiliations:** 1 Department of Endocrinology and Diabetes Perth Children's Hospital Perth Australia; 2 Telethon Kids Institute Children's Diabetes Centre The University of Western Australia Perth Australia; 3 Division of Pediatrics The University of Western Australia Perth Australia; 4 Exercise and Health School of Human Sciences The University of Western Australia Perth Australia

**Keywords:** mobile health app, exercise, acT1ve, type 1 diabetes, young people, blood glucose level

## Abstract

**Background:**

Empowering young people with type 1 diabetes (T1D) to manage their blood glucose levels during exercise is a complex challenge faced by health care professionals due to the unpredictable nature of exercise and its effect on blood glucose levels. Mobile health (mHealth) apps would be useful as a decision-support aid to effectively contextualize a blood glucose result and take appropriate action to optimize glucose levels during and after exercise. A novel mHealth app acT1ve was recently developed, based on expert consensus exercise guidelines, to provide real-time support for young people with T1D during exercise.

**Objective:**

Our aim was to pilot acT1ve in a free-living setting to assess its acceptability and functionality, and gather feedback on the user experience before testing it in a larger clinical trial.

**Methods:**

A prospective single-arm mixed method design was used. Ten participants with T1D (mean age 17.7 years, SD 4.2 years; mean HbA_1c_, 54 mmol/mol, SD 5.5 mmol/mol [7.1%, SD 0.5%]) had acT1ve installed on their phones, and were asked to use the app to guide their exercise management for 6 weeks. At the end of 6 weeks, participants completed both a semistructured interview and the user Mobile Application Rating Scale (uMARS). All semistructured interviews were transcribed. Thematic analysis was conducted whereby interview transcripts were independently analyzed by 2 researchers to uncover important and relevant themes. The uMARS was scored for 4 quality subscales (engagement, functionality, esthetics, and information), and a total quality score was obtained from the weighted average of the 4 subscales. Scores for the 4 objective subscales were determined by the mean score of each of its individual questions. The perceived impact and subjective quality of acT1ve for each participant were calculated by averaging the scores of their related questions, but were not considered in the total quality score. All scores have a maximal possible value of 5, and they are presented as medians, IQRs, and ranges.

**Results:**

The main themes arising from the interview analysis were “increased knowledge,” “increased confidence to exercise,” and “suitability” for people who were less engaged in exercise. The uMARS scores for acT1ve were high (out of 5) for its total quality (median 4.3, IQR 4.2-4.6), engagement (median 3.9, IQR 3.6-4.2), functionality (median 4.8, IQR 4.5-4.8), information (median 4.6, IQR 4.5-4.8), esthetics (median 4.3, IQR 4.0-4.7), subjective quality (median 4.0, IQR 3.8-4.2), and perceived impact (median 4.3, IQR 3.6-4.5).

**Conclusions:**

The acT1ve app is functional and acceptable, with a high user satisfaction. The efficacy and safety of this app will be tested in a randomized controlled trial in the next phase of this study.

**Trial Registration:**

Australian New Zealand Clinical Trials Registry (ANZCTR) ACTRN12619001414101; https://www.anzctr.org.au/Trial/Registration/TrialReview.aspx?id=378373

## Introduction

Managing blood glucose levels during and after exercise is challenging for young people with type 1 diabetes (T1D). Despite the many physical and psychological health benefits of regular exercise, many individuals do not meet physical activity recommendations of at least 60 min/day of moderate to vigorous activity [1,2]. A recent survey based on self- and parent-report revealed that 28% of youth with T1D aged 9 to 17 years were insufficiently active [3]. Among the multiple barriers to engaging in a physically active lifestyle, T1D-specific major barriers include fear of hypoglycemia and insufficient knowledge of managing diabetes around exercise [4-6]. Empowering individuals with T1D to manage their blood glucose levels during exercise is a complex challenge faced by health care professionals, as many factors can influence an individual’s glycemic response to exercise, such as exercise type, intensity, and duration [7,8]; fitness levels; insulinemic state [9]; environmental conditions; and anxiety and stress levels [10]. Technological advances in diabetes management, such as insulin pumps and continuous glucose monitoring (CGM) systems, have aided in the management of diabetes during and after exercise for people with T1D [8]; however, managing the level of blood glucose around physical activity remains one of the biggest challenges to overcome due to the often unpredictable nature of exercise and its effect on blood glucose levels [11].

Key professional societies and organizations have published recommendations for the prevention of exercise-related hypoglycemia based on previous clinical studies and expert opinions [12-14]. However, these recommendations can be challenging to follow, and are often located in medical journals and not readily accessible to the general T1D community and clinicians alike. In addition, health care professionals believe that the lack of formal education in exercise metabolism, and limited time and resources to do so are common barriers to providing guidance around exercise management [15]. Therefore, adolescents and young adults with T1D may benefit from having access to decision-support aids to effectively contextualize a blood glucose result and take appropriate action to optimize glucose levels during and after exercise.

Current care models provide very limited physical activity support to people with T1D. Previous research conducted by our team proposed that providing exercise guidelines in a mobile health (mHealth) app would be useful as a decision-support aid around exercise management for adolescents and young adults with T1D [6]. Global use of mHealth apps is on an exponential rise, and these tools provide a useful platform to deliver health behavior interventions [16-18]. In particular, mHealth apps for diabetes self-management are promising and proliferating at a very high rate [17,18], since diabetes (and in particular T1D) is well suited to smartphone-based support given the use of technology around glucose monitoring, insulin dosing, and carbohydrate counting.

Recent reviews of the literature [19-25] revealed that insulin/medication recording features were found frequently in these apps, as were carbohydrate logs, diet recording features, and physical activity tracking features. Personalized feedback or advice based on patient data, typically insulin dosage suggestions, was also available in 17% of the apps reviewed [21]. However, personalized education is an underrepresented feature, with none of these apps providing personalized advice around exercise.

T1D mobile phone–based interventions hold great promise, but few studies have shown definitive proof of improved health outcomes in this population [26]. Recently, improvement in glucose monitoring and significant improvement in HbA_1c_ [27] in youth with T1D have been reported with the Bant app, which provides personalized feedback by tracking meals, blood glucose, physical activity, and weight data, and is designed primarily for young people with T1D. Since mHealth apps can provide real-time support to their users in addition to traditional clinical counselling, we attempted to develop an exercise app to address the exercise management needs of adolescents and young adults with T1D.

**Figure 1:**
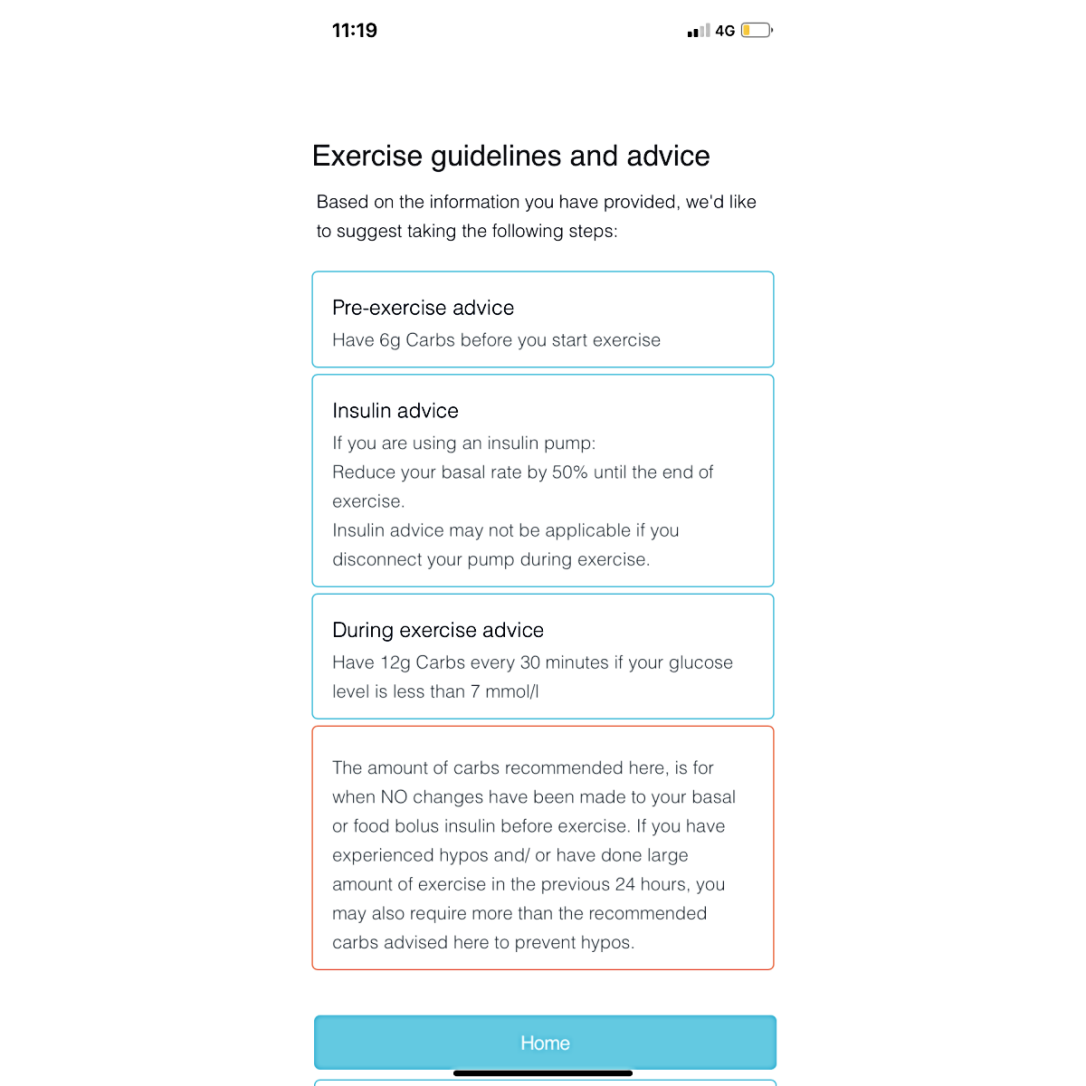
Example screenshot from acT1ve of the carbohydrate and insulin dose recommendation based on information provided by the participant.

Based on recent consensus exercise guidelines [12-14], “acT1ve,” a novel mHealth app, was developed in collaboration with researchers, young people with T1D, and the digital health company Curve Tomorrow, according to a user-centered design (UCD) process that engages end users to ensure app effectiveness [28]. acT1ve uses an exercise advisor algorithm developed in house, consisting of 240 possible pathways depending on user inputs. Participants are prompted to answer questions about the type, intensity, and duration of physical activity they are about to complete; duration since the last insulin bolus; and their current blood glucose levels, with this information then used to provide them with a personalized insulin dose and carbohydrate advice for exercise lasting up to 60 minutes (Figure 1). In addition, acT1ve provides more information on hypoglycemia treatment, pre-exercise and postexercise insulin and carbohydrate advice, and an educational food guide that highlights the importance of low and high glycemic index foods in the context of exercise management.

The aim of this study was to pilot acT1ve in a free-living setting with adolescents and young adults with T1D to assess its acceptability and functionality and to gather feedback to improve the user experience of the app before testing it in a larger clinical trial. Here, mainly viewed from a technological perspective, this study served the following two purposes: (1) to determine whether the app is usable and accepted by users; and (2) to test the deployment of the app before it is rolled out for a larger clinical trial.

## Methods

### Participants

Adolescents and young adults who were aged 12 to 25 years with a diagnosis of T1D for more than 6 months, were on multiple daily injections (MDIs) or continuous subcutaneous insulin infusion (CSII), were exercising regularly (at least twice per week), were competent in English, and had a smartphone were eligible to participate. Recruitment was performed through the Western Australian Children’s Diabetes Database and Perth Children’s Hospital diabetes clinics. The study was advertised on websites and social media. Eligible participants were provided with study information at their clinic visits and via email. Participants provided consent in accordance with the Child and Adolescent Health Human Research Ethics Committee, registered with the National Health and Medical Research Council’s Australian Health Ethics Committee. Parental consent was also obtained for participants under the age of 18 years. The goal of the recruitment was to have enough participants till saturation was attained. Recruitment ceased when saturation was achieved with 10 participants. 

### Methods and Measures

This study was based on a mixed methods prospective single-arm pilot design to assess the acceptability of the acT1ve intervention. Participants visited the research facility on 2 occasions. On the first occasion, the following demographic and descriptive characteristics of the participants were collected: age, sex, duration of diabetes, HbA_1c_, insulin therapy, and exercise patterns. Then, participants were shown how to install acT1ve onto their smartphones. Once installed, participants set up their profile and followed the in-built on-boarding process that guided them through the different sections and functions of acT1ve before leaving the research facility.

Participants were then advised to continue exercising (at least) twice a week over the following 6 weeks with acT1ve, but were otherwise free to use the app as they pleased for their exercise management. Once per week during this 6-week period, the study coordinator contacted study participants and/or their parents/guardians to ensure that the participants were not experiencing more hypoglycemia than usual, remind them to use acT1ve and exercise regularly, troubleshoot any technical problems, and obtain any interim comments or feedback that participants may have had. Study data and survey responses were collected and managed using institutional review board–approved Research Electronic Data Capture (REDCap) [29].

### User Mobile Application Rating Scale Survey

At the end of this 6-week period, participants returned to the research facility and completed the user Mobile Application Rating Scale (uMARS) survey [30]. The uMARS is used to assess the overall quality of mHealth apps, and provides a 20-item measure that includes 4 objective quality subscales, namely engagement, functionality, esthetics, and information quality, and 1 subjective quality subscale. Another subscale, consisting of 6 items, is added to measure users’ perceived impact of the evaluated app [30]. Responses are scored on a 5-point Likert-type scale (1, inadequate; 2, poor; 3, acceptable; 4, good; 5, excellent) [31]. A score of 3 or above was considered acceptable. More specifically, the subscales are as follows: engagement subscale with 5 items assessing entertainment, interest, customization, interactivity, and target group appeal; functionality subscale with 4 items assessing app performance, ease of use, navigation, and gestural design; esthetics subscale with 3 items measuring layout, graphics, and visual appeal; information subscale with 4 items measuring quality and quantity of the written and visual information in addition to the credibility of the source; subjective quality measure with 4 items assessing recommendation and usage of the app, payment, and star rating; and perceived impact measure with 6 items assessing the awareness of the importance of exercise management, an increase in knowledge/understanding of blood glucose management, attitudes toward improving this health behavior, an increase in intention/motivation to address this health behavior, a change in health behavior, and the encouragement of help-seeking behavior, should the participant need it.

### Interview

Participants lastly completed an interview that consisted of both structured and semistructured questions. The interview questions (Multimedia Appendix 1) were designed to gain an understanding of participants’ experiences during the study period to determine if and how any key aspects of acT1ve could be improved, and to investigate whether the use of the app resulted in any improvements in their enjoyment, confidence, frequency, and duration of exercise. Additionally, participants were asked about their overall impressions of the app, and whether they would use it again and recommend it to their peers. All interviews were audio recorded for transcription and analysis. The app was deleted from each participant’s phone at the end of the study period.

### Statistical Analysis

#### Qualitative Analysis

Two qualitative approaches were utilized. Qualitative content analysis was used deductively to analyze the responses to the structured questions, which asked directly about changes in exercise frequency and enjoyment, app useability and acceptability, app recommendation to others, and suggestions for improvement of the app features. This process involves reading and reviewing the data to identify and quantify the content and explore usage rather than meaning. Open coding and categories were created to explain the data [32]. Additionally, thematic analysis was used inductively to identify and report patterns throughout the interview transcripts and determine themes, according to the coding framework outlined by Braun and Clark [33]. Interview transcripts were independently read and reread by 2 researchers, important and relevant codes were identified, and the codes were further explored and clustered to develop themes to explain the data. Any discrepancies were discussed with the research team until a consensus was reached.

#### Quantitative Analysis

The uMARS was scored for the 4 quality subscales described above (engagement, functionality, esthetics, and information), and a total quality score was obtained from the weighted average of the 4 subscales. Scores for the 4 objective subscales were determined by the mean score of each of the individual questions. The perceived impact and subjective quality of acT1ve for each participant were calculated by averaging the scores of their related questions, but were not considered in the total quality score. All scores have a maximal possible value of 5, and they are presented as medians, IQRs, and ranges.

## Results

### Demographics

Ten individuals (8 females and 2 males) were enrolled in this study. They had a mean age of 17.7 (SD 4.2) years, T1D duration of 7.2 (SD 4.8) years, and HbA_1c_ of 54 (SD 5.5) mmol/mol (7.1%, SD 0.5%), and engaged in physical activity for 4.5 (SD 2.9) hours per week. Five participants used CSII and 5 used MDIs. Seven of the 10 participants used a CGM system to monitor their glucose levels. Among the 10 participants, 5 had prior experience with using an exercise-based mobile app; however, none of these were diabetes specific. All 10 participants had acT1ve installed on an Apple iPhone. No participants stopped using the app before the end of the 6-week period.

Weekly contacts with the participants provided information on technical issues and higher than normal hypoglycemia experienced by participants. Two participants experienced a technical error when recording feedback on the app on 1 occasion only. Of the 10 participants, only 2 reported hypoglycemia events after exercise related to app use. One participant followed only 1 of the 2 insulin strategies suggested by the app after exercise. The other participant, who would normally remove the pump while running, continued to use the pump with a reduction in the basal insulin rate as suggested by the app and experienced hypoglycemia.

### Qualitative Analysis: Thematic

**Figure 2:**
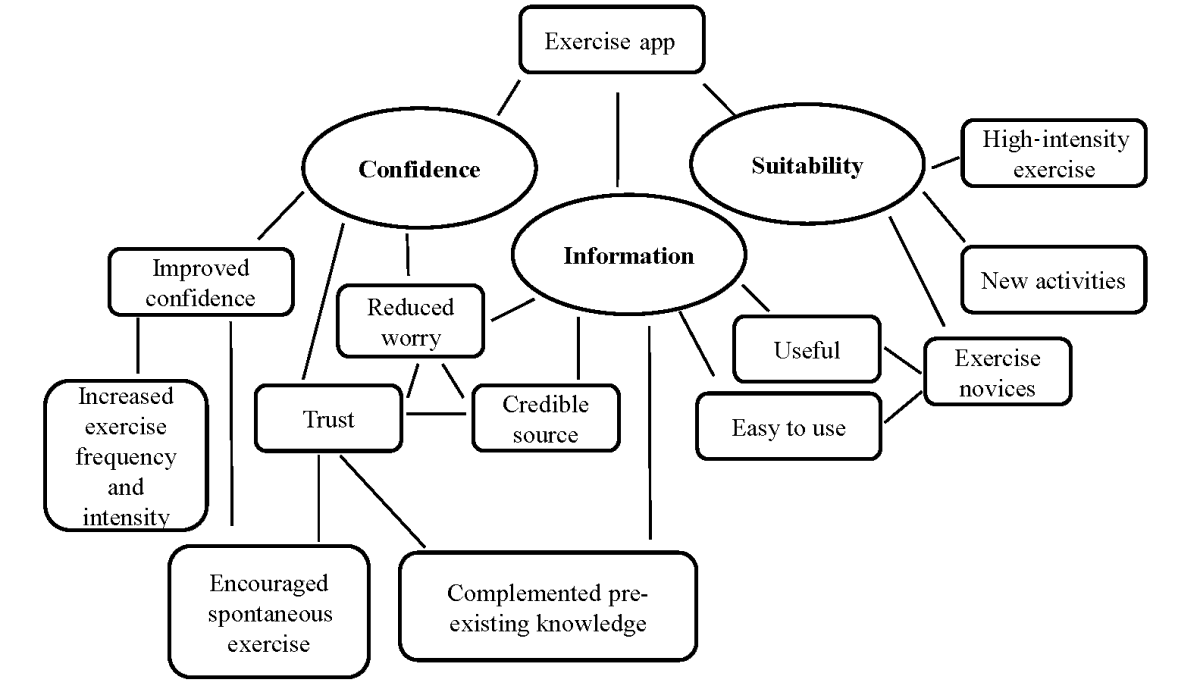
Thematic map of the interview analysis showing 3 overarching themes and the interrelation of subthemes.

The 3 main themes identified from the postintervention interview transcripts were “increased knowledge” (information), “increased confidence to exercise” (confidence), and “suitability for people who are less engaged in exercise” (suitability). These 3 themes and interrelated subthemes are shown in the thematic map (Figure 2), and are discussed below, accompanied by selective illustrative quotations.

#### Information

Some participants commented that the specific information they received from the app was useful in providing new practical information and was credible.

I was like, ok so I should be having this much to eat before, ok, reduce basal here, and stuff like that. Guidance was helpful.

Participant #10

I think the main thing that I really liked was taking into account how much insulin you’ve got on board, how long ago was your bolus, that’s the thing that people really use and really like.

Participant #2

It gave me information that I hadn’t (previously) been given.

Participant #9

I knew the advice it was giving me was legitimate and from like doctors and stuff.

Participant #5

Some participants noted how the information enabled them to keep stable blood glucose levels when physically active.

Normally I would be going inside for a low treatment every 10 minutes, but I was barely going low when I had the app.

Participant #7

Participants also commented on how the information provided by the app complemented their pre-existing knowledge.

You pick and choose or use their information, take it your own way. Which I think is really good as well, because people should come up with their own things.

Participant #2

While most participants found the information they received from the app useful, some individuals commented that the amount of information provided was too much to read and absorb, the information did not always work for them, or the information was repetitive.

Fun to use, but a lot of info, a lot of reading, which I’m happy to do, but it is a bit of information overload.

Participant #1

#### Confidence

The second theme identified was related to the feeling of increased confidence to exercise while using the app. Participants began to trust the information they received from the app, which contributed to improve confidence and lessen worry, particularly in relation to hypoglycemia while physically active.

Before having this app, I would hesitate before exercising, just because I was worried about my level because I didn’t feel as confident in like knowing how much to eat or how giving insulin would affect my levels after exercise, but using this app just gave me a bit more confidence.

Participant #3

After I worked out, using the advice the first time, it worked really well for me. So I knew that I didn’t have to worry about going low during the exercise and even afterwards, I know I’d have a good sleep.

Participant #4

I felt like I didn’t need to guess, and felt more assured in what I was doing to prepare for exercise; because information was coming from the app, it was probably going to work.

Participant #3

One participant (participant #1) commented that using the app was like “having a security blanket.”

Participants stated they became more confident with the app the more they used it, and this led to the participants learning to trust the app over time.

At the start, I would switch between following and not following [the advice provided], but towards the end I always followed it.

Participant #7

#### Suitability

With respect to app suitability for people who are less engaged in exercise, participants commented on the types of activities and who they thought the app would be especially useful for. Some participants acknowledged it being useful mainly for high-intensity activity or when undertaking a new activity.

I’d use it for things that are new. Once I’ve used it enough, and get an idea, I probably wouldn’t have to use it. But I probably would still use it just to make sure I’m doing the right thing.

Participant #5

The app would be particularly suitable for people who aren’t really into exercise yet or who have the fear of what happens if they go low during or after physical activity.

Participant #4

…it might be useful for other people who “struggle with sport”.

Participant #9

### Qualitative Analysis: Content

Content analysis was used to explore specific predetermined items, namely exercise frequency and enjoyment; app useability and acceptability; recommendation of the app to others; and suggestions for improvement of the app.

#### Exercise Frequency and Enjoyment

Six participants commented that using the app had encouraged them to increase their amount of exercise, engage in more spontaneous exercise, and/or increase the intensity of their usual exercise regime during the 6-week study period.

I don’t think I did anything new, just did more of what I usually do [more intense/longer].

Participant #3

While 1 participant commented that exercise was more enjoyable while using the app “because it took the stress away” (participant #4)*,* the other participants reported that their enjoyment of exercise had not changed during app usage.

It made me want to exercise, which is good, I think it was more comfortable to exercise rather than more enjoyable.

Participant #2

#### Useability and Acceptability

All participants felt that acT1ve was generally straightforward and easy to use, with 4 of the 10 participants feeling confident after the first time of use. Other participants had to use the app on several occasions to become confident with it, with 9 of the participants acknowledging that there were enough instructions within the app to get started.

Very easy and straight forward. I had no issues with it at all in regards to finding where I had to go.

Participant #1

Just easy to use and worked 90% of the time.

Participant #7

Participants felt that the convenience of having the app’s information on a phone was excellent, with 3 participants choosing to use acT1ve in conjunction with their own methods of glycemic management, using the app as a guide.

#### Recommendation

Overall, acT1ve was well liked by participants, who all stated that they would recommend it to friends and other people with T1D, and suggested that it might also help others such as teachers or sport coaches.

100% I have a lot of friends in the same boat as me, so it will be a hit I’m sure.

Participant #1

I’d recommend to heaps of people. Even just having an exercise app, everyone wants one just to use, and its good specifically type 1. So it you can log exercise, look at what you’ve done, but it’s nice that it’s just for my diabetes as well.

Participant #2

Participants reported that they would use it again themselves if it was available.

Why? Because it’s a good app, with good information that worked for me.

Participant #8

#### Suggestions for Improving the App

The interview also gave participants the opportunity to provide valuable feedback and suggestions for improving the app prior to its roll out for a larger trial. Their suggestions included a help section on how to use the app for those who may need extra guidance, added information for longer duration of exercise, minimization and simplification of some of the information, suggestions for data sharing and social interaction features, and suggestions for improving esthetics, including more color, personalization for notifications, profile pictures, and *emojis*.

Really good, gives a lot of information and will help with exercise and give people confidence. Wouldn’t say 5 because it can be confusing sometimes, could see people having an issue or rushing through information and not really reading it. Lack of color is a minus, especially for kids, is something that should be incorporated.

Participant #2

I like the app and it had good information, I just think it could do with more personalization on the user’s behalf, eg, choosing your own emoji, and notifying you with how much exercise you’ve done, with more visuals.

Participant #8

### Quantitative Analysis

#### Sports Logged by Participants

Over 6 weeks, participants used acT1ve to obtain exercise management advice for their sports/activities 134 times in total (mean 13.4, SD 7.2 times per participant). Walking, running, and team sports accounted for just over half (51%, 68/134) of the activities logged by participants. Swimming, cycling, and strength training accounted for 18% (24/134) of the activities logged. Other activities like group workout, vaulting, cardio, dance, pilates, yoga, rock climbing, golf, athletics, and skipping accounted for the remaining 31% (42/134) of the activities logged.

#### uMARS 

**Figure 3:**
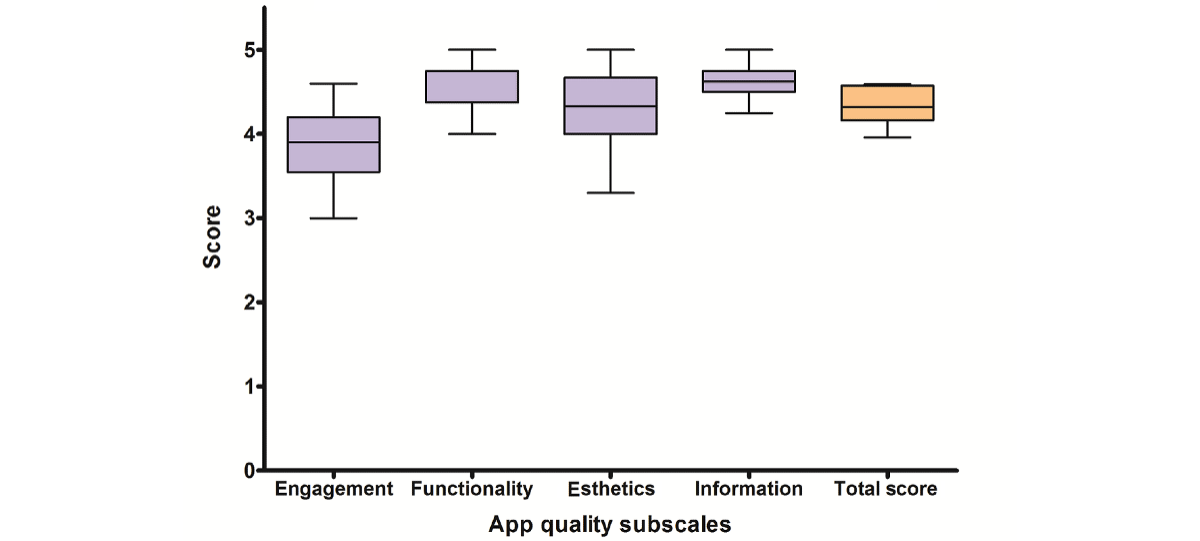
Aggregate participant (n=10) scores (median, IQR, and range) of the 4 user Mobile Application Rating Scale quality subscales (engagement, functionality, esthetics, and information) and the total quality score.

The uMARS total quality median score (out of 5) was 4.3 (IQR 4.2-4.6) (Figure 3), and the objective quality subscale scores were 3.9 (IQR 3.6-4.2) for engagement, 4.8 (IQR 4.5-4.8) for functionality, 4.3 (IQR 4.0-4.7) for esthetics, and 4.6 (IQR 4.5-4.8) for information (Table 1). The median scores for all subjective quality items were above 4 (Table 2), with the exception of “payment” (asks participants how likely they are to pay for acT1ve). The median scores for all perceived impact items (where a score of 1=strongly disagree and a score of 5=strongly agree) were 4 or above (Table 2), with the exception of “knowledge,” with a score of 3.5 (IQR 3.2-4.6). Lower scoring items were customization (median 3.0, IQR 3.0-3.0) in the engagement subscale and payment (median 3.0, IQR 2.0-3.8) in the subjective quality measure.

**Table 1:** Objective quality and total quality scores in the user Mobile Application Rating Scale (uMARS) evaluation.

Measure, subscale, and item				Score, median (IQR)
**Objective quality**				
	**Engagement**			3.9 (3.6-4.2)
		Entertainment	4.0 (3.3-4.0)	
		Interest	4.0 (4.0-4.8)	
		Customization	3.0 (3.0-3.0)	
		Interactivity	4.0 (4.0-4.0)	
		Target group appeal	4.0 (4.0-5.0)	
	**Functionality**			4.8 (4.5-4.8)
		Performance	5.0 (5.0-5.0)	
		Ease of use	4.0 (4.0-5.0)	
		Navigation	5.0 (4.0-5.0)	
		Gestural design	5.0 (4.0-5.0)	
	**Esthetics**			4.3 (4.0-4.7)
		Layout	5.0 (4.0-5.0)	
		Graphics	5.0 (4.2-5.0)	
		Visual appeal	4.0 (3.3-4.0)	
	**Information**			4.6 (4.5-4.8)
		Quality information	5.0 (4.0-5.0)	
		Quantity information	4.5 (4.0-5.0)	
		Visual information	4.5 (4.0-5.0)	
		Credibility	5.0 (5.0-5.0)	
Total quality				4.3 (4.2-4.6)

**Table 2:** Subjective quality and perceived impact scores in the user Mobile Application Rating Scale (uMARS) evaluation.

Measure and item		Score, median (IQR)
**Subjective quality**		4.0 (3.8-4.2)
	Recommend	5.0 (4.0-5.0)
	Usage	4.5 (4.0-4.8)
	Payment	3.0 (2.0-3.8)
	Star rating	4.0 (4.0-4.8)
**Perceived impact**		4.3 (4.0-5.0)
	Awareness	4.0 (4.5-4.8)
	Knowledge	3.5 (3.0-5.0)
	Attitudes	4.5 (3.3-5.0)
	Intention to change	4.0 (4.0-4.8)
	Help seeking	4.0 (4.0-4.8)
	Behavioral change	4.0 (3.3-5.0)

## Discussion

### Principal Findings

mHealth apps can provide real-time support to users, in addition to traditional clinical counselling. In order to find out if mHealth apps can provide people with T1D with real-time support during exercise, we piloted acT1ve, a novel mHealth app, to assess its acceptability and functionality, and gather feedback to improve the user experience of the app before testing it in a larger clinical trial. We found that acT1ve was functional and acceptable, with high user satisfaction.

The qualitative and quantitative analyses of this study provided important insights into the perspectives of participants in relation to the functionality and usability of the app. The information provided by the app was found to be relevant, appropriate, and clear, with a simple and easy flow of presentation. Participants felt they received adequate information to guide their diabetes management and enable them to maintain stable blood glucose levels during physical activity. This reduced their worry about their glucose levels and provided them with trust and confidence to be more physically active. Trust was gained because the information came from a credible source and complemented their pre-existing knowledge.

Many participants felt the information received from the app was beneficial, and acknowledged that it would be helpful for high-intensity exercise, when undertaking a new activity, for people struggling with sports, and for those who have fear of exercise-related hypoglycemia. These findings are not surprising since these situations are very challenging for exercise management in T1D. Indeed, the unpredictability of glycemic responses during a new activity and the variable glycemic response during and after high-intensity activities depending on the prevailing insulin levels [34,35] increase the complexity of exercise management [12-14]. Hence, having an exercise advisor app available during physical activities was reported to be useful. Unlike the diabetes education information currently available in approximately 35% of apps accessible for diabetes self-management [25], acT1ve provides real-time decision support around exercise. As reported by Lum et al [36], of the approximately 370 diabetes apps that met the researchers’ criteria for blood glucose self-management, the majority did not provide real-time decision support or situation-specific education on blood glucose self-management. Only 10% of apps educated users on blood glucose management [36], and none educated users on maintaining stable glycemia around exercise.

acT1ve was found to be engaging, informative, and functional with appropriate esthetics. The participants liked the design of the app and found it acceptable and useful. They indicated that they would likely continue to use it long term and also recommend it to friends and other people with T1D. It has been established that for a user to adopt and frequently use a smartphone app long term, the user must consider it both usable and useful [37,38]. A recent review [39] examining evidence supporting commercially available apps for diabetes self-management and a detailed assessment of app features, privacy/security, and usability found variable results on app usability. Of the 5 apps available for usability testing for T1D, 1 was acceptable, 3 were marginal, and 1 was not acceptable [39]. Though these results suggest that patients may have had a difficult time using some of these apps, our results are not comparable, since usability was assessed not by patients using the app, but by reviewers rating each available app using the System Usability Scale (SUS), which includes 10 Likert-like items [40]. They used guidance from Bangor et al [41] to interpret SUS ratings (≥70 B, acceptable; 50-69 B, marginal; <50 B, not acceptable). Chavez et al [42] used uMARS to analyze the 89 most popular free English language diabetes apps and found that while this subset of mHealth apps ranked “acceptable-good” in engagement, functionality, and esthetics, they ranked “poor-acceptable” in information, app quality, and app subjective. In contrast, acT1ve received high scores for each of the uMARS subscales and its overall quality. The 2 lower scoring items were customization in the engagement criteria and payment in the subjective quality criteria. The lack of customization features in acT1ve settings and preferences that end users would have liked, for example, sound, content, and notifications, needs improvement in the future iterations of acT1ve. Moreover, individuals would be more likely to use acT1ve if it is freely available.

acT1ve has great scope to be a promising tool to support exercise management for youth with T1D since the assessment of practices around exercise in these individuals has shown that there is a lack of understanding, awareness, and adherence to clinical recommendations around exercise [43]. Despite several studies showing an increase in the frequency of hypoglycemia during and after exercise, many youth are not adjusting insulin for exercise [43]. In a recent study, MacMillan et al [44] examined patients’, parents’, and providers’ perceptions of physical activity support in youth with T1D, and found that all of them spoke of limited physical activity encouragement in the current care model. For this reason, they proposed interventions that included education to “build confidence in the patient to participate in physical activity” and inclusion of technology in the interventions to provide in-person support [44]. The use of a diabetes-related smartphone app as an adjunct to usual care combined with weekly text message support from a health care professional has been shown to significantly improve glycemic levels in adults with T1D [45].

The recent consensus report from the joint European Association for the Study of Diabetes and the American Diabetes Association Diabetes Technology Working Group highlights the potential value of digital apps for diabetes self-management [46]. Though an exercise advisor app for T1D has recently been designed [47] and there are increasing numbers of apps designed to give guidance to patients with T1D during exercise [48], to our knowledge, the benefits of a smartphone app have not been tested with respect to its effectiveness at supporting diabetes self-management around exercise in adolescents and young adults with T1D. Our results show that acT1ve has the potential to facilitate glycemia management during exercise and to support the needs of youth with T1D by providing personalized guidance on insulin dosing and carbohydrate intake strategies, and by improving their knowledge and confidence around exercise management.

### Limitations

Despite its many benefits, there are some limitations with acT1ve. For instance, acT1ve was tested only on Apple iOS; however, consumers use a variety of mobile technological platforms. While acT1ve was also compatible with Android devices, iOS appeared to be the dominant operating system in the target group of the study. Another limitation was that even though this app was developed by adopting the UCD process, acT1ve did not include all the features recommended by the end users in the design process due to budget limitations. This was evident in the uMARS evaluation, where a lower score was given for elements in the engagement criteria like customization and participant feedback for improvement of the app. Some of the suggestions like the help section on how to use the app for those who may need extra guidance, the shortening and simplification of some of the information it provides to avoid confusion, and the improvement of esthetics have all been considered in the amended version of the app. Other desirable features like activity tracker, data sharing options, integration with real-time CGM, social engagement, and interactions with other technological tools like music apps could have further improved its interactivity and will be considered in future iterations of the app. Since the scores from the uMARS do not necessarily reflect the real impact in terms of behavior change and health outcomes, further studies are needed to assess the efficacy, safety, and clinical significance of acT1ve for diabetes self-management around exercise.

Since the aim of this study was to test the usability and acceptability of the app, only patients who were exercising at least two or more times per week were enrolled in the study without accounting for the wide range of HbA_1c_, different activity levels of patients, or their barriers to exercise. All these variables will be addressed by conducting a randomized controlled trial in the next phase of this study.

### Conclusions

This study suggests that our novel mHealth app acT1ve is informative, functional, and acceptable, and that users were satisfied with using it. Our app may thus provide a promising intervention for exercise management for adolescents and young individuals with T1D. The app was well received by all users and was found to be simple to use with easy-to-follow advice and smooth functioning. The end users reported less anxiety about exercising, knowing credible advice was readily available. Self-management is the key to diabetes care, and managing glucose levels around exercise has been an ongoing challenge for young people with T1D. Our findings suggest that acT1ve may be a valuable addition or supplement to diabetes management around exercise for adolescents and young adults with T1D. However, additional work is needed to assess the efficacy, safety, and clinical significance of this app.

## Appendix

Multimedia Appendix 1 Interview questions.

## Abbreviations

CGM: continuous glucose monitoring
CSII: continuous subcutaneous insulin infusion
MDIs: multiple daily injections
mHealth: mobile health
SUS: System Usability Scale
T1D: type 1 diabetes
UCD: user-centered design
uMARS: user Mobile Application Rating Scale
