# Supplementary material for: A Novel Mobile Health App to Educate and Empower Young People With Type 1 Diabetes to Exercise Safely: Prospective Single-Arm Mixed Methods Pilot Study
Source: JMIR Diabetes. 2021 Oct 14;6(4):e29739. doi: 10.2196/29739 (PMC8554675; doi:10.2196/29739)
Supplement: Multimedia Appendix 1 [file diabetes_v6i4e29739_app1.docx]

**Multimedia Appendix 1: Interview questions.**

**Introducing the Interview**

- Now that you have used the Exercise APP we would like to hear about what you **liked and didn’t like** about the APP, what you thought of the **features,** and if you think you would **use it**.
- For the purposes of this interview, **exercise** relates to **activity requiring physical effort**, for example, team sports, sport classes, gym sessions, walking to school, mowing the lawn etc.
- There are no right or wrong answers
- The recorded information will not be identifiable, we will not use any names
- This information will be audio recorded but will remain confidential
- Your participation is voluntary so we can stop the interview at any time
- You can choose not to answer any of the questions
- This will take about 20 minutes; you can take a break at any time
- The recorder can be turned off at any time you ask, or parts of the recording can be removed
- Do you have any questions for me?

**Post Trial Questions**

**How often did you exercise during the study period including times you didn’t use the APP?**

- What types of exercise did you most commonly participate in? (type, duration, intensity)

**Was exercise more or less enjoyable using the APP compared to your regular management?**

- - On a scale of 1 to 5 where 1 = exercise was less enjoyable; 3 = about the same enjoyment; 5 = exercise was more enjoyable
- So you picked a number (**) – can you tell me why you didn’t give it a higher/ lower score?

**Did you exercise more or less with the APP compared to your regular management?**

- On a scale of 1 to 5 where 1 = exercised less with the APP; 3 = exercised about the same amount; 5 = exercised more with the APP
- Did you exercise more often, for a longer duration, or both?
- Did the APP allow more freedom to exercise spontaneously?
- Did you participate in different types and intensities of sport?

**Were you more or less confident to exercise with the APP compared to your regular management?**

- - On a scale of 1 to 5 where 1 = you were less confident; 3 = about the same level of confidence; 5 = you were more confident
- So you picked a number (**) – can you tell me why you didn’t give it a higher/ lower score?

**Did you understand how to use the APP?**

- On a scale of 1 to 5 where 1 = I did not understand it....... 5 = I did understand
- So you picked a number (**) – can you tell me why you didn’t give it a higher/ lower score?
- Did you feel confident using the APP?
- How long did it take you to feel confident using the app?
- Were there enough prompts or instructions?
- Looking back what would you have liked to have known from the start?

**How much did you trust the APP?**

- On a scale of 1 to 5 where 1 = did not trust the APP at all; 5 = completely trusted the APP
- How often did you exercise with the APP?
- When you did not use it, why didn’t you use it?
  - - Prompts: was it due to not being suitable for the sport or because you did not trust it?

**How often did you follow the advice the APP gave?**

- On a scale of 1 to 5 where 1 = did not follow the advice at all; 5 = always followed the advice
- On the times you chose to do something else, why was this?

**Was the information you entered into the APP accurate?**

- On a scale of 1 to 5 where 1 = never accurate at all; 5 = always accurate
  - So you picked a number (**) – can you tell me why you chose this score?

**______________________________________________________________**

*I am now going to go through the APP with you to get feedback on individual sections.*** Refer to screenshot documents*

**Did you stop using the APP before the end of the 6 weeks?**

- If YES: What was the reason you stopped using it?

**Did the weekly contact encourage you to use the app?**

- If YES: Explain why.

**Overall what did you think of the APP?**

- On a scale of 1 to 5 where 1 = it’s awful…………… 5 = it’s great
- So you picked a number (**) – can you tell me why you didn’t give it a higher/ lower score?

**How likely are you to recommend this APP to others with type 1 diabetes?**

- On a scale of 1 to 5 where 1= not likely …… 5 = very likely
  - Explore score

**If you had the opportunity to use the APP again,**

- Would you use it?
  - - - How long do you think you would use it for?
      - Would you use it all the time or just during certain times/situations?
      - If during certain times/situations, what are these?

**Ending the interview**

These were all the questions I had for you. Do you have any question for me or anything you would like to add to this interview?

Thank you for taking part.
